# The Chemically-Modified Tetracycline COL-3 and Its Parent Compound Doxycycline Prevent Microglial Inflammatory Responses by Reducing Glucose-Mediated Oxidative Stress

**DOI:** 10.3390/cells10082163

**Published:** 2021-08-22

**Authors:** Nilson Carlos Ferreira Junior, Maurício dos Santos Pereira, Nour Francis, Paola Ramirez, Paula Martorell, Florencia González-Lizarraga, Bruno Figadère, Rosana Chehin, Elaine Del Bel, Rita Raisman-Vozari, Patrick Pierre Michel

**Affiliations:** 1 Sorbonne Université, Paris Brain Institute-ICM, Inserm, CNRS, APHP, Hôpital de la Pitié Salpêtrière, 75013 Paris, France; juniormb@usp.br (N.C.F.J.); msp_biomed@yahoo.com.br (M.d.S.P.); francis.nour@icm-institute.org (N.F.); paola.ramirez@icm-institute.org (P.R.); martorell.paula@icm-institute.org (P.M.); 2 Department of Basic and Oral Biology, FORP, Campus USP, University of São Paulo, Av. Café, s/no, Ribeirão Preto 14040-904, Brazil; eadelbel@usp.br; 3 USP, Center for Interdisciplinary Research on Applied Neurosciences (NAPNA), São Paulo 05508-220, Brazil; 4 Instituto de Investigación en Medicina Molecular y Celular Aplicada (IMMCA) (CONICET-UNT-SIPROSA), CP 4000 Tucumán, Argentina; mflorenciagl@hotmail.com.ar (F.G.-L.); rosanachehin@gmail.com (R.C.); 5 BioCIS, CNRS, Université Paris-Saclay, 92290 Châtenay-Malabry, France; bruno.figadere@universite-paris-saclay.fr

**Keywords:** COL-3, glucose metabolism, microglia, NADPH oxidase, neuroinflammation, oxidative stress, tetracyclines

## Abstract

We used mouse microglial cells in culture activated by lipopolysaccharide (LPS) or α-synuclein amyloid aggregates (αSa) to study the anti-inflammatory effects of COL-3, a tetracycline derivative without antimicrobial activity. Under LPS or αSa stimulation, COL-3 (10, 20 µM) efficiently repressed the induction of the microglial activation marker protein *Iba*-*1* and the stimulated-release of the pro-inflammatory cytokine TNF-α. COL-3′s inhibitory effects on TNF-α were reproduced by the tetracycline antibiotic doxycycline (DOX; 50 µM), the glucocorticoid dexamethasone, and apocynin (APO), an inhibitor of the superoxide-producing enzyme NADPH oxidase. This last observation suggested that COL-3 and DOX might also operate themselves by restraining oxidative stress-mediated signaling events. Quantitative measurement of intracellular reactive oxygen species (ROS) levels revealed that COL-3 and DOX were indeed as effective as APO in reducing oxidative stress and TNF-α release in activated microglia. ROS inhibition with COL-3 or DOX occurred together with a reduction of microglial glucose accumulation and NADPH synthesis. This suggested that COL-3 and DOX might reduce microglial oxidative burst activity by limiting the glucose-dependent synthesis of NADPH, the requisite substrate for NADPH oxidase. Coherent with this possibility, the glycolysis inhibitor 2-deoxy-D-glucose reproduced the immunosuppressive action of COL-3 and DOX in activated microglia. Overall, we propose that COL-3 and its parent compound DOX exert anti-inflammatory effects in microglial cells by inhibiting glucose-dependent ROS production. These effects might be strengthened by the intrinsic antioxidant properties of DOX and COL-3 in a self-reinforcing manner.

## 1. Introduction

Tetracyclines are a family of antibiotics that inhibit bacterial protein synthesis by attaching to the ribosomal subunit and consequently blocking the binding of aminoacyl-tRNAs to the ribosome A site [1]. Although the effects of tetracyclines as antibiotics have long been known, there has been a renewed interest in this family of molecules. Indeed, in addition to their antibacterial activity, they can exert pharmacological effects that may have clinical applications in various disease states, including cancer [2], inflammation-related disorders [3], and CNS neurodegenerative pathologies [4,5]. 

As an illustration of that, doxycycline (DOX), an old tetracycline antibiotic currently used for the treatment of skin problems [6], combines anti-tumoral [7], anti-inflammatory [8], and neuroprotective [9,10] activities. In particular, a number of studies reported neuroprotective effects of DOX in toxin-induced animal models of Parkinson’s disease (PD) [9,10]. DOX was also found to decrease the expression of several inflammation markers in microglial cultures activated with the bacterial inflammogen lipopolysaccharide (LPS) [8], which indicates that DOX’s neuroprotective effects may be due in part to its ability to limit inflammation-related events [9,10]. The anti-inflammatory properties of DOX may also explain why this tetracycline provided relief against L-DOPA-induced dyskinesia in a PD rat model [11]. Interestingly, DOX was also found capable of preventing amyloid aggregation of α Synuclein (αS) and tau, two seeding-prone proteins involved in PD [12,13] and Alzheimer’s disease [14] pathologies, respectively, suggesting that this tetracycline has the potential of a multimodal neuroprotective drug.

**Figure 1:**
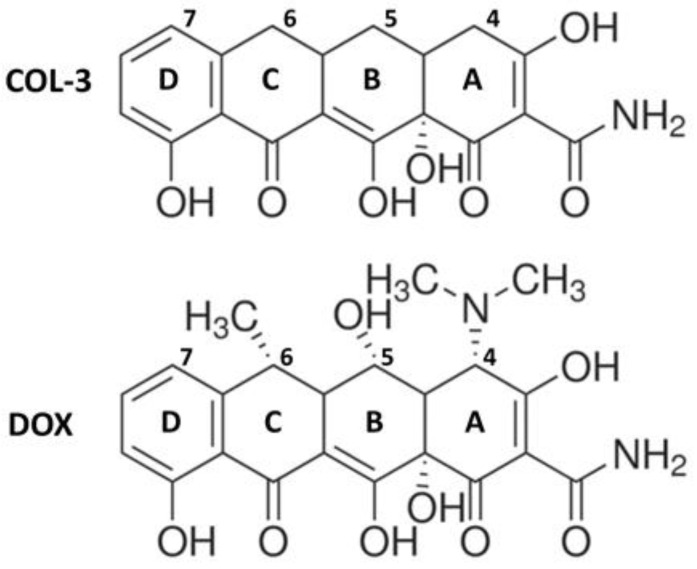
Chemical structures of the non-antibiotic tetracycline COL-3 and of its parent compound DOX. COL-3 differs from DOX by the absence of the DCBA *naphthacene* core structure of dimethylamino-, hydroxyl-, and methyl- groups in C-4, C-5, and C-6, respectively. The loss of antimicrobial activity specifically results from the removal of the dimethylamino group at the C4 position.

COL-3, also named chemically modified tetracycline 3 (CMT-3), 4-dedimethylaminosancycline, or incyclinide, belongs to a group of modified tetracyclines that have been structurally rearranged to suppress their antibacterial properties. Removal of the antimicrobial activity obtained by eliminating the dimethylamino group from carbon 4 in the A ring of the tetracycline core structure (Figure 1) was carried out with the principle of preserving other activities of interest [15,16]. COL-3 has been tested in clinical trials in cancer patients [17,18], and its capacity of inhibiting matrix metalloproteinases offers the possibility to reduce the excessive breakdown of connective tissue in many pathological conditions [19,20,21]. COL-3 is highly lipophilic, so it can cross the blood–brain barrier and exert CNS effects [3,22]. In that respect, systemic administration of COL-3 was reported to inhibit brain microglial activation induced by the bacterial inflammogen LPS [22]. However, the mechanism through which COL-3 reduces neuroinflammation is still unclear. 

In the present study, we wished to further explore the mechanisms underlying the anti-inflammatory activity of COL-3 towards brain microglial cells, using its parent compound DOX as a reference tetracycline molecule. For this research project, we used brain microglial cells challenged with LPS and amyloid aggregates of the synaptic protein αSa in order to model brain inflammatory-type reactions as they may occur in PD neurodegeneration [23,24,25]. We found that COL-3 was intrinsically more potent than DOX, as it was optimally effective at lower concentrations than its parent compound. Both COL-3 and DOX seemed to operate by limiting the capacity of microglia of generating glucose-dependent NADPH and, as a consequence, reactive oxygen species (ROS) via NADPH oxidase activation.

## 2. Materials and Methods

### 2.1. Drugs and Cell Culture Reagents

Lipopolysaccharide (LPS; *Escherichia coli* strain O26:B6; #L8274), 2-deoxy-D-glucose (2-DG; #D8375), Trolox (TROL; #238813), dexamethasone (DEX; #D4902), and DOX hyclate (#D9891) were obtained from Sigma-Aldrich (L’Isle d’Abeau Chesnes, France). COL-3, also named CMT-3, 4-dedimethyl aminosancycline, or incyclinide (#HY-13648), was purchased from MedChemExpress (Monmouth Junction, NJ, USA) and apocynin (APO; #4663) from R&D Systems Europe (Lille, France). Dulbecco’s modified Eagle’s medium (DMEM) and trypsin (0.05%)-EDTA solution, used to generate microglial subcultures, were obtained from ThermoFisher Scientific (Saint Aubin, France). Fetal calf serum (FCS) was provided by Biowest LLC (Eurobio, Les Ulis, France), and 2-[1,2-3H (N)]-deoxy-D-glucose (NET549A) was purchased from Perkin Elmer (Courtaboeuf, France). 

### 2.2. Microglial Cell Cultures

#### Ethics Statement

To generate microglial cell cultures, we used newborn C57BL/6J mice obtained from Janvier LABS (Le Genest St. Isle, France). Mice were kept and cared for, following European Union Council Directives (2010/63/EU). The Committee on the Ethics of Animal Experiments Charles Darwin no. 5 approved experimental protocols. 

### 2.3. Polyethyleneimine Coating

Polyethyleneimine (PEI; Mw: 750,000; Mn: 60,000; #P3143, Sigma-Aldrich, L’Isle d’Abeau Chesnes, France) was applied to culture flasks, as previously described [26]. PEI was diluted in a 40 mM borate buffer solution (1 mg/mL; pH 8.3) prepared with borax decahydrate (#B3545) [26,27]. After 2 h of incubation (37 °C), PEI was discarded, and culture flasks washed four times with Dulbecco’s phosphate-buffered saline (PBS) were kept aside until further use.

### 2.4. Isolation of Microglial Cells and Production of Microglial Subcultures

The brains of mouse pups (postnatal day 1) were mechanically dissociated, as previously described [26]. Microglial cells in suspension were plated in PEI-coated T-75 culture flasks (Corning) containing 12 mL of DMEM supplemented with FCS (10%) and a penicillin/streptomycin cocktail. Under these conditions, microglial cell isolation occurs spontaneously after 14–18 days of culture. When required, purified microglial cells were maintained for up to 1 more week in culture flasks by adding 2–3 mL of DMEM supplemented with penicillin/streptomycin and only 1% of FCS [28]. Subcultures were produced by trypsinizing purified microglial cell cultures with 0.05% trypsin-EDTA for 5 min. After trypsin inactivation with 10% FCS, cells were triturated and plated onto uncoated Nunc 48-multiwell plates (10^5^ cells per well), using DMEM supplemented with antibiotics and 1% FCS as maintenance medium.

### 2.5. Purification and Aggregation of Recombinant αS

The expression and purification of recombinant human αS were performed as previously described [12,29]. The purity of recombinant αS was evaluated by SDS-PAGE, and contaminating endotoxins were removed from protein samples using a Pierce Spin column (#88275; Thermo Fisher Scientific, Courtaboeuf, France). The Limulus amebocyte lysate assay revealed that monomeric αS stock solutions contain less than 0.1 endotoxin units (EU)/mL at this stage. The protein solutions were filtered and centrifuged, and the absorbance of the supernatant was measured at 280 nm using a Nanodrop 8000 spectrophotometer (Thermo Fisher Scientific, Courtaboeuf, France). Finally, protein solutions were adjusted to obtain appropriate working concentrations.

To generate amyloid aggregates of αS, we used a protocol reported by us before [12,30]. Briefly, αS samples incubated at 37 °C were submitted to continuous orbital agitation for 96 h (Thermomixer Comfort; Eppendorf, Montesson, France). Recovered samples sonicated for 2 min with a Branson B3510-DTH ultrasonic bath (VWR International, Fontenay sous Bois, France) were then kept at −20 °C until further use [13]. 

### 2.6. Transmission Electron Microscopy (TEM)

We used TEM characterization to confirm that the process of αS fibrillization occurred in agitated samples [30]. Briefly, samples were adsorbed onto glow-discharged 200-mesh-carbon-film-coated copper grids. Then, samples were stained with 2% uranyl acetate. The liquid in excess was discarded, and the grids were left to dry at room temperature. TEM images were captured using a Hitachi HT7700 120 kV system.

### 2.7. Stimulation Protocols with Inflammogens and Drug Treatments

Microglial cells were treated with either LPS or αSa for 24 h to model brain inflammatory-type reactions. Treatments with test compounds were initiated 2 h before adding the inflammogens to the cultures and prolonged until the end of experimental protocols. Note that none of the test treatments exerted toxic effects on microglial cells under these conditions.

### 2.8. Immunofluorescence Detection of Microglial Cell Markers

A MAC-1/CD11b rat monoclonal antibody (clone M1/70.15) from BioRad (Oxford, UK) and a rabbit antibody raised against ionized calcium-binding adaptor molecule-1 (Iba-1, #019-19741) from Wako Chemicals (Neuss, Germany) were used to monitor microglial cell responses, at the cellular level. Briefly, we fixed microglial cells in 4% formaldehyde in PBS (20 min at room temperature) and then incubated them sequentially with antibodies against MAC-1/CD11b (1:100 in PBS for 72 h) and Iba-1 (1:500 in PBS 0.2% Triton X-100 overnight). The immunodetection of MAC-1/CD11b and Iba-1 was performed with anti-rat Alexa-Fluor 488- and anti-rabbit Alexa-Fluor 555-conjugated antibodies, respectively (Invitrogen, Waltham, MA, USA). Nuclear counterstaining with the cell-permeable Hoechst-33342 dye (1 μg/mL for 5 min) was performed to enable automated focus for quantitative image analysis protocols (Arrayscan XTi workstation; Thermo Fisher Scientific, Courtaboeuf, France). Fluorescent images were acquired with a 20× objective, and variations in immunofluorescence signal intensities were quantified using the HCStudio software (Thermo Fisher Scientific, Courtaboeuf, France). Approximately 2500 microglial cells were analyzed for each treatment condition.

### 2.9. TNF-α Assay

TNF-α was quantified using an ELISA kit from Thermo Fisher Scientific (Courtaboeuf, France) (#BMS607-3) according to the manufacturer’s protocol. Absorbance was measured at a wavelength of 450 nm using a SpectraMax i3X microplate reader (Molecular Devices, Sunnyvale, CA, USA). 

### 2.10. Measurement of Intracellular NADPH

A high-sensitivity NADPH quantitation fluorometric assay kit (#MAK216; Sigma-Aldrich, L’Isle d’Abeau Chesnes, France) was used to assess intracellular NADPH levels, as previously described [31]. The fluorescent reaction product was evaluated with a SpectraMax i3X microplate reader (Molecular Devices, Sunnyvale, CA, USA) using an excitation wavelength of 535 nm and an emission wavelength of 587 nm.

### 2.11. Quantification of Intracellular ROS Levels

Intracellular ROS levels were measured using the CellROX Deep Red reagent (Invitrogen Life Technologies, Waltham, MA, USA), as previously described [31]. Signal acquisition was performed on an Arrayscan XTI automated workstation fitted with a 20× objective (Thermo Fisher Scientific, Courtaboeuf, France). Cellular fluorescence intensity was estimated using HCStudio software.

### 2.12. Assessment of [^3^H]-2-DG Uptake

[^3^H]-2-DG uptake was assessed as previously described [31]. Briefly, after termination of test treatments, microglial cell cultures were incubated at 37 °C for 30 min in PBS containing 1 μCi [^3^H]-2-DG. 2-DG accumulation was interrupted by adding ice-cold PBS to the cultures. Microglial cells in culture were then scrapped in a 1% Triton-X–distilled water solution, and the radioactivity accumulated intracellularly was measured using a Tri-Carb 4910TR liquid scintillation counter (Villebon Sur Yvette, France). Blank values were measured in control wells containing 50 mM glucose.

### 2.13. Statistical Analysis

Data values were collected from a minimum of two independent experiments, each performed in duplicate cultures. Experimental data are presented as the mean ± SEM. Statistical analyses were performed using one-way ANOVA followed by a Bonferroni post-hoc test using the GraphPad Prism version 8 for Windows (GraphPad Software, La Jolla, CA, USA).

## 3. Results

### 3.1. COL-3 Reduces the Inflammatory Response of Microglial Cells Exposed to LPS or αSa

**Figure 2:**
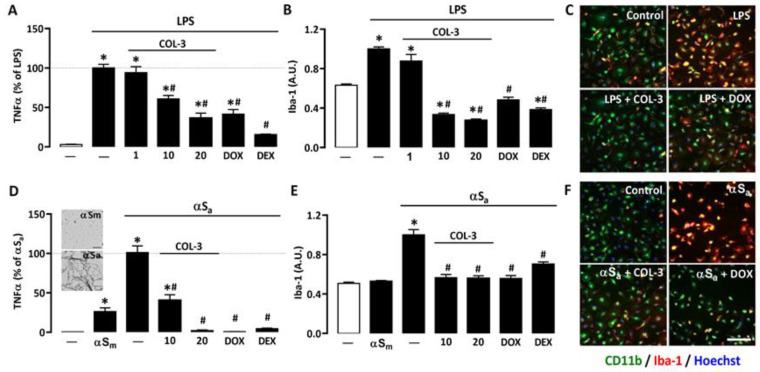
COL-3 reduces inflammatory responses in microglial cells exposed to either LPS or αSa. (**A**) TNF-α levels in microglial cell cultures exposed to LPS (10 ng/mL, 24 h) in the presence or absence of COL-3 (1, 10, or 20 μM), DOX (50 µM), or the reference anti-inflammatory drug DEX (2.5 μM). Data are the mean ± SEM (*n* = 4). * *p* < 0.05 vs. control; ^#^ *p* < 0.05 vs. LPS. One-way ANOVA followed by Bonferroni post-hoc test. (**B**) Quantification of Iba-1 immunofluorescence signals in microglial cell cultures treated as in (**A**). (**C**) Merged images of CD11b (green) and Iba-1 (red) immunofluorescence signals in LPS (10 ng/mL)-treated microglial cell cultures receiving COL-3 (20 µM) or DOX (50 µM). The reddish/yellow fluorescence denotes overexpression of Iba-1. Cell nuclei are counterstained with Hoechst-33342 (blue). (**D**) TNF-α levels in microglial cell cultures treated with αSa (70 µg/mL, 24 h) in the presence or absence of COL-3 (10 and 20 μM), DOX (50 µM), or DEX (2.5 μM). Some cultures were also exposed to αSm (70 µg/mL). Data are the mean ± SEM (*n* = 4). * *p* < 0.05 vs. control; ^#^ *p* < 0.05 vs. αSa. One-way ANOVA followed by Bonferroni post-hoc test. TEM images from the insert show the presence of αS amyloid fibrils in αS samples shaken for 96 h (lower image) and the absence of aggregated species in non-shaken samples (upper image). Scale bar = 1 μm. (**E**) Quantification of Iba-1 immunofluorescence signals in microglial cultures exposed to the same treatments as in (**D**). Data are the mean ± SEM (*n* = 4). * *p* < 0.05 vs. control; ^#^ *p* < 0.05 vs. αSa. One-way ANOVA followed by Bonferroni post-hoc test. (**F**) Merged images of CD11b (green) and Iba-1 (red) immunofluorescent signals in αSa (70 µg/mL)-treated microglial cell cultures receiving COL-3 (20 µM) or DOX (50 µM). The reddish/yellow fluorescence denotes overexpression of Iba-1. Cell nuclei are counterstained with Hoechst-33342 (blue). Scale bar = 120 µm. Data are the mean ± SEM (*n* = 4). * *p* < 0.05 vs. control; ^#^ *p* < 0.05 vs. LPS or αSa. One-way ANOVA followed by Bonferroni post-hoc test.

To assess the anti-inflammatory potential of the non-antibiotic tetracycline COL-3, we used primary microglial cell cultures treated with LPS (10 ng/mL), a prototypical agonist of Toll-like receptor (TLR)4 [32,33]. After 24 h of LPS exposure, the production/release of the pro-inflammatory cytokine TNF-α was strongly increased. In cultures treated with 10 or 20 μM COL-3, TNF-α production was reduced by more than 40% and 70%, respectively (Figure 2A). No significant inhibitory effect was observed, however, at 1 µM, indicating that the anti-inflammatory action of COL-3 was concentration-dependent and only significant at 10 µM or above. At 20 µM, COL-3 appeared to be as effective as the reference tetracycline DOX at 50 µM. The reference anti-inflammatory drug DEX at 2.5 µM was more effective than COL-3 at 20 µM or DOX at 50 µM in reducing TNF-α release.

We also performed immunosignal intensity measurements for the phenotypic activation marker Iba-1 in the same experimental conditions. As expected, Iba-1 expression was significantly up-regulated in LPS-treated cultures. COL-3 suppressed Iba-1 induction at 10 and 20 µM but was without significant effect, at 1 µM (Figure 2B). DOX (50 µM) also efficiently reduced the Iba-1 immunofluorescence signal in LPS-treated cultures. Note that 2.5 µM DEX reduced Iba-1 expression with an efficacy similar to that of 20 µM COL-3 or 50 µM DOX. Photomicrographs in Figure 2C illustrate the impact of COL-3 (20 µM) or DOX (50 µM) treatments on Iba-1 immunosignal intensities in LPS-treated microglial cells immunostained with CD11b.

To model inflammatory reactions as they may occur in PD [23,24], we used αSa to trigger an inflammogenic response in microglial cell cultures. The illustration from the insert of Figure 2D confirms the presence of amyloid fibrils in αS samples shaken for 96 h (lower image), whereas non-shaken samples are free of such species (upper image). Figure 2D shows that 24 h of exposure to 70 µg/mL αSa results in a robust increase in TNF-α release, whereas the monomeric form of the protein (αSm) induced only a limited but significant response in microglial cells. At 10 or 20 µM, COL-3 significantly reduced αSa-induced TNF-α release. DOX at 50 µM exerted a comparable repressive effect, while 2.5 µM DEX was as effective as 20 µM COL-3 and 50 µM DOX.

Consistent with previous observations [24], we found that αSa induced Iba-1 overexpression. COL-3 (10 or 20 µM), DOX (50 µM), and DEX (2.5 µM) were all effective in reducing the Iba-1 immunofluorescent signal in αSa-treated microglial cultures (Figure 2E). Note that the intensity of the Iba-1 immunosignal was not modified by αSm despite the fact that TNF-α release was slightly increased by such treatment. This may signify that quantifying TNF-α with an ELISA assay kit may be more sensitive than measuring changes in cellular Iba-1 expression levels when microglial inflammatory responses are of small intensity. Photomicrographs in Figure 2F represent microglial cell cultures treated with αSa in the presence or absence of COL-3 (20 µM) or DOX (50 µM), and then immunostained for CD11b and Iba-1.

### 3.2. COL-3 Reduces Glucose Accumulation in Microglial Cells Exposed to LPS or αSa

**Figure 3:**
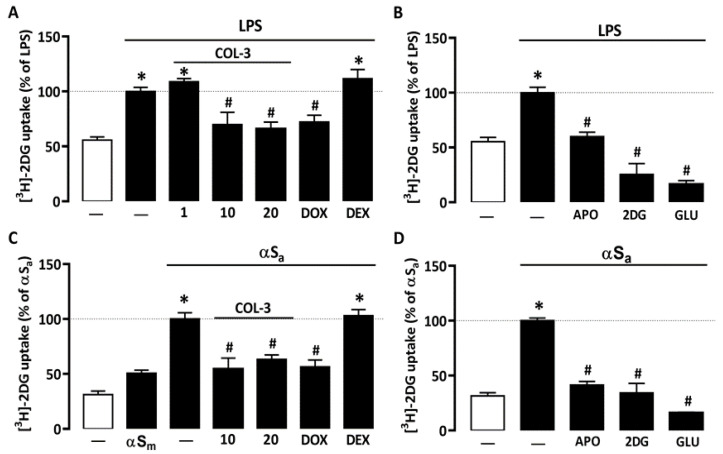
COL-3 reduces glucose accumulation in microglial cells exposed to either LPS or αSa. (**A**) Quantification of glucose uptake in microglial cells exposed or not to LPS (10 ng/mL, 24 h) in the presence or absence of COL-3 (10 or 20 μM), DOX (50 µM), or DEX (2.5 μM). (**B**) Quantification of glucose uptake in microglial cells exposed or not to LPS treatment in the presence or absence of APO (300 µM) or 2-DG (500 µM). The specificity of the assay was confirmed by acutely exposing LPS-treated cell cultures to an excess of glucose (50 mM). (**C**) Quantification of glucose uptake in microglial cells exposed or not to αSa (70 µg/mL, 24 h) in the presence or absence of COL-3 (10 and 20 μM), DOX (50 µM), or DEX (2.5 μM). (**D**) Quantification of glucose uptake in microglial cells exposed or not to αSa in the presence or absence of APO (300 µM) or 2-DG (500 µM). The specificity of the assay was confirmed by acutely exposing αSa-treated cell cultures to an excess of glucose (50 mM). Data are the mean ± SEM (*n* = 6–8). * *p* < 0.05 vs. control; ^#^ *p* < 0.05 vs. corresponding inflammogen. One-way ANOVA followed by Bonferroni post-hoc test.

Recent studies suggest that microglial inflammatory responses are profoundly dependent on glucose availability [31,34,35]. Here, we found that 24 h of treatment with 10 ng/mL of LPS led to an almost 2-fold increase in [^3^H]-2-DG uptake in microglial cell cultures (Figure 3A). [^3^H]-2-DG uptake was significantly reduced when LPS-treated cell cultures were exposed to either 10 or 20 µM COL-3, whereas a lower COL-3 concentration (1 µM) had no significant effect on this parameter (Figure 3A). DOX at 50 µM was also effective in reducing microglial glucose accumulation. Noticeably, DEX at 2.5 µM had no impact on [^3^H]-2-DG uptake, indicating that this compound may exert immunosuppressive effects in microglial cells without affecting glucose metabolism. In addition, 300 µM of the NADPH oxidase inhibitor APO or 500 µM of unlabeled 2-DG [a concentration not affecting microglial cell viability], efficiently reduced [^3^H]-2-DG uptake in agreement with previous observations [31]. As expected, 50 mM glucose added acutely to microglial cell cultures to compete with [^3^H]-2-DG during uptake assessment also efficiently prevented [^3^H]-2-DG uptake (Figure 3B).

Interestingly, 70 µg/mL αSa also significantly increased [^3^H]-2-DG uptake in microglial cell cultures (Figure 3C), suggesting that the inflammatory response of microglial cells to αS fibril exposure is also fueled by glucose. By contrast, αSm had only a marginal impact on glucose consumption. Noticeably, COL-3 (10 or 20 µM) significantly reduced glucose accumulation in cultures exposed to αSa; DOX at 50 µM mimicked this effect. As described previously for LPS, DEX (2.5 µM) failed to inhibit TNFα release stimulation by αSa. The treatment of αSa-treated cultures with 300 µM APO or 500 µM 2-DG also led to a significant reduction in [^3^H]-2-DG uptake (Figure 3D). Adding glucose in excess when performing uptake measurements also prevented [^3^H]-2-DG uptake (Figure 3D).

### 3.3. COL-3 Prevents the Rise of NADPH in Microglial Cells Exposed to LPS or αSa

**Figure 4:**
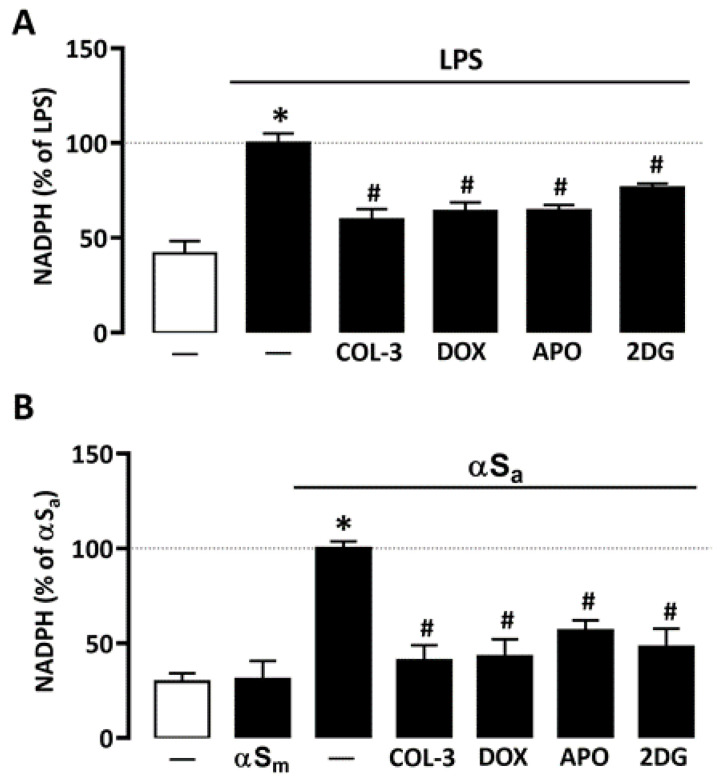
COL-3 prevents NADPH synthesis in microglial cells exposed to either LPS or αSa. (**A**) Quantification of NADPH in microglial cells exposed or not to LPS (10 ng/mL, 24 h) in the presence or absence of COL-3 (20 μM), DOX (50 µM), APO (300 µM), or 2-DG (500 µM). (**B**) Quantification of NADPH in microglial cells exposed or not to αSa (70 µg/mL, 24 h) in the presence or absence of COL-3 (20 μM), DOX (50 µM), APO (300 µM), or 2-DG (500 µM). Data are the mean ± SEM (*n* = 6–8). * *p* < 0.05 vs. control; ^#^ *p* < 0.05 vs. corresponding inflammogen. One-way ANOVA followed by Bonferroni post-hoc test.

Glucose may have a key impact on microglial cell inflammatory processes by activation of the pentose phosphate pathway and, consequently, by stimulating NADPH synthesis, a required substrate for the superoxide-producing enzyme NADPH oxidase [31,34]. Consistent with this view, NADPH levels rose significantly in microglial cells treated with either 10 ng/mL of LPS or 70 µg/mL αSa (Figure 4A,B). No effect was observed, however, with αSm. Interestingly, COL-3 (20 µM) significantly reduced NADPH levels in both inflammation paradigms (Figure 4A,B). As expected, the inhibitory effects of COL-3 in each activation paradigm were mimicked with DOX at 50 µM.

Both 2-DG (500 µM) and the NADPH oxidase inhibitor APO (300 µM) mimicked the inhibitory effect of COL-3 on NADPH production in microglial cells exposed to LPS or αSa (Figure 4A,B), indicating that the non-antibiotic tetracycline and its parent compound DOX may interfere with a glucose-dependent mechanism that promotes NADPH synthesis and, consequently, ROS production via NADPH oxidase activation.

### 3.4. COL-3 Prevents Microglial Oxidative Stress Elicited by Inflammatory Signals

**Figure 5:**
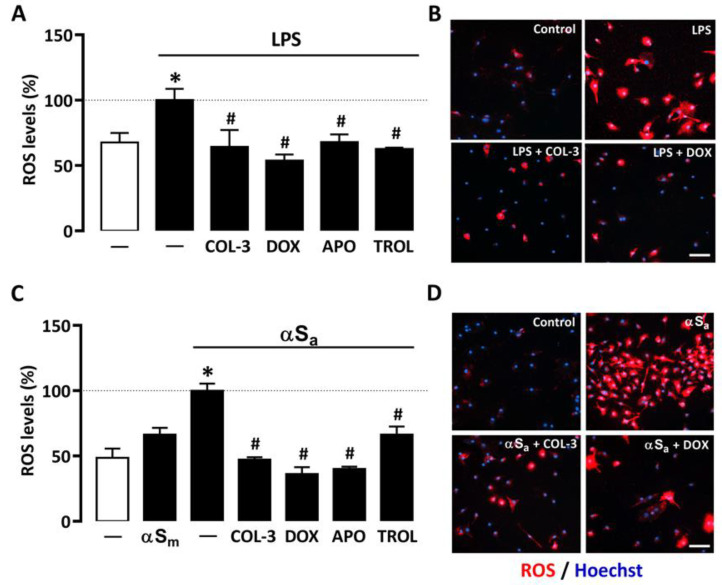
COL-3 prevents intracellular microglial oxidative stress generated by LPS or αSa exposure. (**A**) Quantification of ROS levels in microglial cells exposed or not to LPS (10 ng/mL, 24 h) in the presence or absence of COL-3 (20 μM), DOX (50 µM), APO (300 µM), or TROL (10 µM). Data are the mean ± SEM (*n* = 6–8). * *p* < 0.05 vs. control; ^#^ *p* < 0.05 vs. LPS. One-way ANOVA followed by Bonferroni post-hoc test. (**B**) Visualization of intracellular ROS levels in LPS (10 ng/mL)-treated microglial cell cultures receiving or not COL-3 (20 µM) or DOX (50 µM). Scale bar = 50 µm. (**C**) Quantification of ROS levels in microglial cells exposed or not to αSa (70 µg/mL, 24 h) in the presence or absence of COL-3 (20 μM), DOX (50 µM), APO (300 µM), or TROL (10 µM). Data are the mean ± SEM (*n* = 6–8). * *p* < 0.05 vs. control; ^#^ *p* < 0.05 vs. αSa. One-way ANOVA followed by Bonferroni post-hoc test. (**D**) Visualization of intracellular ROS levels in αSa (70 µg/mL)-treated microglial cell cultures exposed or not to COL-3 (20 µM) or DOX (50 µM) treatment. Scale bar = 50 µm.

A quantitative cell-based fluorescence assay was performed to estimate ROS levels in microglial cells treated with LPS or αSa and other test treatments (Figure 5). As expected, 24 h of treatment with LPS (10 ng/mL) or αSa (70 µg/mL) significantly increased intracellular ROS levels in microglial cells. By contrast, αSm had no effect. Interestingly, ROS production was maintained near control values when microglial cell cultures exposed to either LPS (Figure 5A,B) or αSa (Figure 5C,D) were treated with 20 µM COL-3, confirming the view that the non-antibiotic tetracycline may exert anti-inflammatory effects by counteracting oxidative-stress-dependent mechanisms. As expected, the inhibitory effect of COL-3 on ROS generation was mimicked by DOX at 50 µM. In each activation paradigm, the NADPH oxidase inhibitor APO (300 µM) and the antioxidant TROL (10 µM) reduced microglial ROS production with an efficacy similar to that demonstrated by COL-3 and DOX. 

### 3.5. The Suppressive Effect of COL-3 on TNF-α Release Is Reproduced by Interfering with Glucose Metabolism or NADPH Oxidase Activation

**Figure 6:**
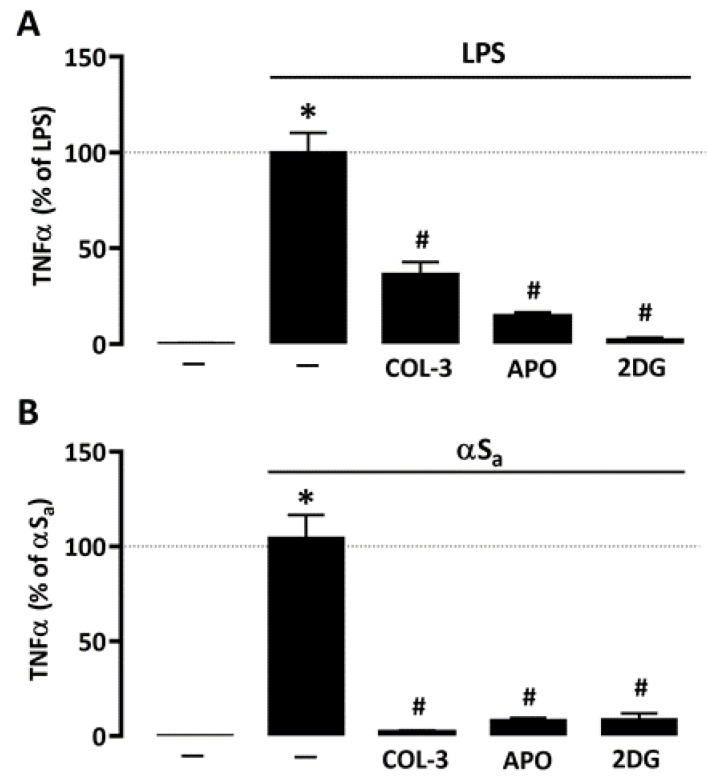
Inhibitory effects of COL-3 on TNF-α release are reproduced by inhibition of either glucose metabolism or NADPH oxidase activity. (**A**) Quantification of TNF-α levels in microglial cell cultures exposed or not to LPS (10 ng/mL, 24 h) in the presence or absence of COL-3 (20 μM), APO (300 µM), or 2-DG (500 µM). Data are the mean ± SEM (*n* = 6). * *p* < 0.05 vs. control; ^#^ *p* < 0.05 vs. LPS. One-way ANOVA followed by Bonferroni post-hoc test. (**B**) Quantification of TNF-α levels in microglial cell cultures exposed or not to αSa (70 µg/mL, 24 h) in the presence or absence of COL-3 (20 μM), APO (300 µM), or 2-DG (500 µM). Data are the mean ± SEM (*n* = 6). * *p* < 0.05 vs. control; ^#^ *p* < 0.05 vs. αSa. One-way ANOVA followed by Bonferroni post-hoc test.

Given the repressive action of COL-3 on microglial glucose consumption and ROS production, we wished to compare its effect to that of 2-DG and APO, using TNF-α release as reference inflammation marker. Similar to COL-3, APO (300 µM) and 2-DG (500 µM) were highly effective in reducing LPS- (Figure 6A) or αSa-induced (Figure 6B) TNF-α release, further supporting the view that COL-3 exerts its anti-inflammatory effect by interfering with a glucose-dependent mechanism that promotes oxidative stress via NADPH oxidase activation (Figure 6B).

## 4. Discussion

COL-3 is a non-antibiotic tetracycline with a number of interesting pharmacological properties, most of them related to its anti-inflammatory and anti-cancer activities. Using microglial cell cultures, we demonstrated, here, that COL-3 and its parent compound DOX can efficiently restrain inflammatory responses triggered by either the bacterial cell wall component LPS or amyloid fibrils of the PD protein αS. COL-3 appeared more potent than its parent compound DOX in mitigating microglial inflammatory responses. The anti-inflammatory potential of COL-3 to limit microglial activation appeared to be closely associated with its capacity to restrain glucose-dependent ROS production. DOX appears to operate in a similar manner.

### 4.1. COL-3 Performs as a Potent Anti-Inflammatory Drug in LPS- and αSa-Treated Microglial Cells

We wished to evaluate the anti-inflammatory potential of COL-3 in microglial cells challenged with two inflammatory triggers, the bacterial cell wall component LPS and amyloid aggregates of the synaptic protein αS. We established that concentrations of COL-3 comprised between 10 and 20 µM were quite effective in limiting microglial inflammatory responses. Specifically, we found that COL-3 reduces the expression of the microglial/macrophage activation marker Iba-1 and the secretion of the pro-inflammatory cytokine TNF-α in both activation paradigms. LPS operating by activation of TLR4 [32] and αSa by stimulation of TLR2/ P2X7 purinergic receptors [24,30], one may assume that COL-3 inhibited signaling events that are common to these two activation pathways. The reference tetracycline DOX was not only effective against LPS-induced inflammation as reported before by us [8], but it also reduced inflammogenic effects of αS fibrils, confirming the view that DOX closely mimicked COL-3′s immunosuppressive action. COL-3 appeared, however, intrinsically more potent than its parent compound DOX in repressing microglial cell responses, as lower concentrations of COL-3 were needed for optimal anti-inflammatory effects. Incidentally, this confirms that the anti-inflammatory potential of tetracyclines is totally dissociated from their antimicrobial properties.

Present findings are also consistent with studies reporting on the effects of COL-3 in experimental models of inflammation. For instance, COL-3 was found to inhibit paclitaxel-induced thermal hyperalgesia [36], a condition with a strong inflammatory component [37]. Cazalis et al. [38] showed that COL-3 was also capable of reducing cytokine secretion in an ex vivo human whole-blood model used to study periodontitis inflammation. In addition, COL-3 was reported to be effective in limiting microglial cell activation and TNF-α (but not IL-1β) production in the brain of mice receiving LPS intraperitoneal injections [22]. 

### 4.2. Anti-Inflammatory Properties of COL-3 Derive from Its Ability to Inhibit Glucose Uptake

Together with others, we reported that inflammatory conditions cause dramatic changes in microglial glucose metabolism [31,34,39]. Precisely, we found that glucose (i.e., 2-DG) uptake was strongly enhanced in microglial cells activated by the bacterial inflammogen LPS [31]. We confirmed here this initial observation and showed that microglial cells responded similarly to αSa, suggesting that changes in glucose metabolism are not restricted to TLR4-dependent mechanisms but also pertain to activation paradigms relying on TLR2/ P2X7 purinergic receptors [24,30]. Remarkably, COL-3 (10–20 µM) robustly inhibited glucose uptake in both LPS- and αSa-treated microglial cells, suggesting that COL-3 may repress both inflammatory responses by reducing glucose utilization. At a concentration of 50 µM, the reference antibiotic tetracycline DOX mimicked the inhibitory effects of COL-3 on glucose uptake, which indicates that a reduction in glucose availability may also contribute to the anti-inflammatory effects of DOX. Incidentally, the fact that glucose uptake was measured after removal of COL-3 and DOX from the cultures indicates that these compounds did not repress glucose accumulation through a simple competitive inhibition mechanism.

Note that DEX failed to repress microglial glucose uptake in activated microglia, regardless of the inflammation trigger. This is unexpected since it has been reported that DEX can inhibit glucose uptake in contracting myotubes [40]. Yet, glucocorticoids classically inhibit the transcription of several genes that encode pro-inflammatory cytokines and chemokines [41], suggesting that alternative mechanisms are involved in DEX-immunosuppressive effects for microglial cells.

### 4.3. COL-3 Prevents Glucose-Dependent NADPH Production

Glucose is known to fuel glycolysis and oxidative phosphorylation through glycolytic pyruvate [42]. In microglial cells, our group and others [31,34] have shown that a substantial fraction of glucose entering glycolysis is diverted at the level of Glucose-6-P to the pentose monophosphate shunt for regenerating NADPH, a dinucleotide operating as a requisite substrate for NADPH oxidase [42,43]. Interestingly, we show here that the increase in glucose consumption induced by LPS and αSa treatments resulted in a rise in NADPH synthesis that is preventable by COL-3. This indicates that the immunosuppressive action of COL-3 observed against the two inflammatory signals may result from its ability to limit the glucose-dependent synthesis of NADPH and, as a consequence, ROS production by NADPH oxidase. As expected, DOX also suppressed NADPH synthesis in microglial cells challenged with LPS and αSa, confirming the view that the two tetracyclines operate in a similar manner to mitigate microglial inflammatory reactions. Even if our results suggest that COL-3 and DOX act principally by reducing NADPH, generated via the pentose phosphate pathway, additional effects of the two tetracyclines on glycolytic enzymes cannot be totally excluded [39].

### 4.4. COL-3 Operates by Preventing Glucose-Dependent ROS Production

The use of the fluorogenic probe CellROX Deep reagent revealed that ROS production was robustly increased in LPS and αSa-activated microglial cells in good agreement with previous reports [24,30,44]. Interestingly, ROS induction was efficiently inhibited by COL-3 and DOX. Both the antioxidant vitamin E derivative TROL and the inhibitor of the ROS producing enzyme NADPH oxidase APO mimicked the suppressive action of COL-3 and DOX on ROS production. This signifies that COL-3 and DOX may operate by preventing a sequence of signaling events in which glucose promotes NADPH oxidase-mediated ROS production and subsequent inflammatory responses such as TNFα production. Coherent with this view, blocking NADPH oxidase activity with APO prevented TNFα release after exposure to LPS or αSa. We may assume that the inhibitory effect of COL-3 on TNFα release resulted from the inhibition of the redox-sensitive nuclear transcription nuclear factor-κB, a master regulator of cytokine production in microglial cells [31,44]. As a matter of fact, DOX was reported previously to inhibit NF-kB activation in different model systems [8,45].

The efficacy of COL-3 and DOX to restrain glucose-dependent NADPH synthesis suggests that both tetracyclines mitigate microglial inflammatory responses by reducing the amount of NADPH available as a cofactor for NADPH oxidase. Pointing to the importance of such a mechanism, we found that restraining glucose metabolism and NADPH synthesis with the non-metabolizable glucose analogue 2-DG was sufficient *per se* to reproduce the suppressive effects of COL-3 and DOX on TNF-α release. Unexpectedly, Fodelianaki and colleagues reported that 2-DG was unable to prevent LPS-induced TNF-α release in microglial cell cultures [39]. The reason for such a discrepancy between the two studies is rather unclear at this stage.

**Figure 7:**
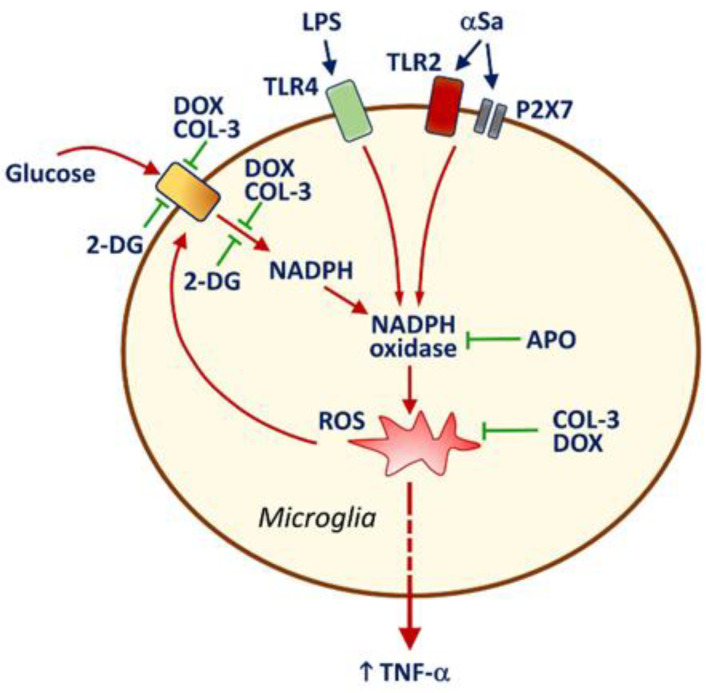
Schematic drawing illustrating how the non-antibiotic tetracycline COL-3 and its parent compound DOX may limit microglial inflammatory responses. LPS and αSa activate TLR4 [33] and TLR2/P2X7 [24,30] signaling pathways, respectively. This results in the activation of the superoxide producing enzyme NADPH oxidase. ROS produced through NADPH oxidase activation stimulate the release of the pro-inflammatory cytokine TNF-α and enhance the uptake of glucose through a mechanism that is preventable by the NADPH oxidase inhibitor APO. The rise in microglial glucose stimulates the synthesis of NADPH through the activation of the pentose phosphate shunt [31]. Increased levels of NADPH, the required substrate for NADPH oxidase [42,43], stimulate ROS production by this enzyme. The inhibition of glucose uptake by COL-3 and DOX leads to a reduction in glucose-derived synthesis of NADPH, which limits ROS production by NADPH oxidase. These effects may be strengthened by intrinsic antioxidant properties of COL-3 and DOX [47] in a self-reinforcing manner. Limiting glucose-dependent NADPH synthesis with the non-metabolizable analogue of glucose, 2-DG restrains microglial inflammatory reactions, indicating that the control of glucose metabolism by COL-3 and DOX is pivotal for their immunosuppressive effects. Note that DEX prevents efficiently TNF-α release but has no inhibitory effect on glucose uptake, suggesting that the glucocorticoid operates through a mechanism of action that is distinct from that of COL-3 or DOX. Inhibitory and activation signals are represented in green and red, respectively.

It is also worth noting that NADPH oxidase inhibition by APO caused a drop in glucose uptake in activated microglia, indicating that ROS might represent an activation signal for microglial glucose accumulation via a specific transport system. As a matter of fact, ROS production by NADPH oxidase was reported to stimulate glucose accumulation via a mechanism implicating glucose transporter-4 in muscle cells [46]. This means that the inhibitory effects of COL-3 and DOX on glucose uptake may also partly depend on the intrinsic antioxidant properties of these two compounds [47,48]. Therefore, one may assume that the capacity of COL-3 and DOX to inhibit glucose-dependent ROS production via a suppressive effect on NADPH synthesis is strengthened by intrinsic antioxidant properties of the two tetracyclines in a self-reinforcing manner. A schematic drawing illustrating how COL-3 and DOX may limit microglial inflammatory responses is provided in Figure 7.

Overall, the present data confirm that the non-antibiotic tetracycline COL-3 and its parent compound DOX can efficiently mitigate microglial inflammatory responses. The anti-inflammatory effects of COL-3 and DOX appear to primarily result from their capacity to restrain glucose-dependent NADPH synthesis and, as a consequence, ROS production via NADPH oxidase activation. Present data provide further confirmation that COL-3 and DOX may have therapeutic utility in chronic CNS pathological conditions such as PD, where long-term neuroinflammatory processes play a contributive role. COL-3 might have, however, an advantage over DOX, as it does not possess antibiotic activity.

## Data Availability

Data will be available when requested.

## Abbreviations

αS	α-synuclein
αSa	αS aggregates
αSm	αS monomers
APO	Apocynin
CMT-3	Chemically modified tetracycline
COL-3	4-dedimethylaminosancycline or incyclinide
2-DG	2-deoxy-D-glucose
DMEM	Dulbecco’s modified Eagle’s medium
DOX	Doxycycline
FCS	Fetal calf serum
Iba-1	ionized calcium-binding adaptor molecule-1
LPS	Lipopolysaccharide
PD	Parkinson’s disease
PEI	Polyethyleneimine
ROS	Reactive oxygen species
TLR	Toll-like receptor
TROL	Trolox
